# Growing of *Phalaroides arundinacea* L. and *Bromopsis inermis* Leyss for Biofuel Using Sewage Sludge Compost as a Fertilizer

**DOI:** 10.3390/plants12233939

**Published:** 2023-11-22

**Authors:** Jelena Ankuda, Eugenija Bakšienė, Almantas Ražukas

**Affiliations:** Vokė Branch, Institute of Agriculture, Lithuanian Research Centre for Agriculture and Forestry, Žalioji Sq. 2, LT-02232 Vilnius, Lithuania

**Keywords:** energy crops, biomass, sewage sludge compost, soil, heavy metals

## Abstract

*Bromopsis inermis* and *Phalaroides arundinacea* are increasingly grown not only for feed but also for other purposes, such as obtaining energy. Composting sewage sludge and using such compost to fertilize various plants is also becoming more popular. Therefore, the aim of our study was to investigate how the fertilization of the mentioned plants with sewage sludge compost (SSC) affects the biomass yield and biomass quality of these plants. It was also important to determine whether fertilization with SSC is possible in low-yielding soils of light texture without harming these soils There are no similar studies in Lithuania, and there are very few in Europe and the world. It was found that investigated grasses formed a small biomass DM yield (6.6 t/ha within three years). The biomass of *B. inermis* and *Ph. arundinacea* had a very high ash content and concentrations of nitrogen and potassium. This greatly reduced the quality of the biomass of these grasses as a solid biofuel. SSC performed poorly as a fertilizer. Fertilization with 125 t/ha SSC significantly increased the total biomass yield of *Ph. arundinacea* only. At the same time, using SSC as a fertilizer significantly increased the concentration of Cu, Zn, and Cd in the soil.

## 1. Introduction

Currently, a strong interest in renewable energy has become a worldwide tendency [1,2,3,4]. This concerns solar, wind, geothermal, water, and biomass energies [2,4]. These energy resources are environmentally friendly and are the main instruments for mitigating climate change [1,2,3,4,5,6,7]. The European Union has stressed the importance of biomass use, proposing a variety of opportunities for development and promoting the use of plant biomass as an energy source as intensively and as widely as possible. In Lithuania, the use of renewable energy sources, on the basis of the regulating legal instruments, is regarded as one of the priority areas of energy development [8].

Energy plants are herbaceous plants and short-rotation energy forests that are grown for their biomass to be used for biofuels, electricity, and thermal energy production [9]. One of the ways to obtain energy from plants is to burn them. It is considered that during plant combustion the release of CO_2_ is equal to that used by plants for photosynthesis [4,10,11]. Consequently, burning the biomass of energy plants is considered an environmentally friendly way of obtaining energy that does not contribute to global climate change. However, not all plant biomass is cost-effective if used as a solid biofuel. The studies that have intended to identify the plants that produce more energy while burning and that cause less damage to boilers are particularly relevant [12,13,14,15]. Additionally, almost a quarter of all Lithuanian total farmland area is unproductive and of low productivity [16,17]. In such lands, traditional farming is economically useless, and growing energy plants could be a good alternative. In addition, growing perennial energy crops could potentially enrich barren soils with organic carbon and reduce soil degradation, erosion, and the leaching of nutrients from the soil [18,19]. All over the world, including in Lithuania, scientists have conducted various studies in order to determine which energy plants outperform others, and which growing conditions are the best for growing biomass suitable for obtaining energy. Some authors claim that short-rotation energy forests (willows (*Salix* spp.), poplars (*Populus* spp.), and others) are the most valuable energy plants because they form a large biomass dry matter (DM) yield and can grow for a very long time in one place [20,21,22]. However, cutting down short-rotation energy forests is problematic and costly. Perennial herbaceous plants can also be grown for a long time in one place. For example, plants of the genus *Miscanthus* can produce high yields for up to 15–20 years while growing in the same place [23,24] and *Bromopsis inermis* L. can grow in one place for up to 30–40 years [25,26]. In addition, the biomass of some perennial grass species can be cut several times during the growing season. Grass biomass is much easier and cheaper to cut than the biomass of short-rotation energy forests. Studies on the cultivation of *Bromopsis inermis* Leyss. and *Phalaroides arundinacea* L. are very relevant because the demand for these plants is increasing. These grasses are increasingly grown not only for fodder but also for other purposes, such as obtaining energy (for biogas production, solid biofuel, etc.). *Bromopsis inermis* Leyss. variety ‘*Galinda*’ and *Phalaroides arundinacea* L. variety ‘*Alaušas*’ were bred in the Vokė branch of the Lithuanian Institute of Agriculture (Vokė Branch of the Institute of Agriculture of the Lithuanian Research Centre for Agriculture and Forestry (LAMMC) at this moment) (authors—J. Šedys [25,27,28] and R. Vaičiulytė [25]) and are best adapted to the light soils and climatic conditions of this area of Lithuania. The plants of these varieties are characterized by a high biomass DM yield.

Currently, along with the increase in population around the world, the world’s food and energy needs are increasing [29,30]. Therefore, the aim is to obtain a higher yield of various plants. As a result, the use of mineral fertilizers has increased. However, too intensive use of mineral fertilizers can harm the soil. Chemical elements (nitrogen, phosphorus, and others) can leach into groundwater and contaminate it and surface waters [30]. Finally, mineral fertilizers are expensive. 

Sewage sludge disposal is an urgent issue at the moment [31]. Improperly handled or neglected sewage sludge poses a threat to the environment and human health. One of the ways to dispose of it, while simultaneously obtaining economic benefits, is composting and using it for the fertilization of energy plants [32,33,34]. In European countries, the fertilization of plants with sewage sludge and its compost is being studied [35,36,37]. Sludge and compost are rich in various elements (N, P, K, Ca, and Mg) and bases that are beneficial to plants; they are also characterized by a neutral pH, which increases the biological activity of soil and humus content and reduces soil bulk density and acidity [38,39,40]. However, sewage sludge compost, as well as sewage sludge, contains high concentrations of heavy metals; therefore, it is especially important to adjust fertilization so that the heavy metals do not adversely affect the environment, including the soil properties and cultivated plants. It has also not been studied at all whether the heavy metals are intensively absorbed from the soil by *B. inermis* and *Ph. arundinacea.* This would be useful to determine in order to find out the possibilities of using these grasses for cleaning heavy metals from the soil because their concentrations would inevitably increase when sewage sludge compost is used as a fertilizer. Furthermore, energy plants fertilized with sewage sludge compost can accumulate particularly high amounts of nitrogen, phosphorus, potassium, chlorine, and other elements in their biomass. It is known that the overall emission rate for biofuel-fired boilers strongly depends not only on the boilers and their control but also on the quality of fuel. For example, the nitrogen and chlorine contents in biomass can be reduced by agrotechnical measures and optimization of plant fertilization [10].

Thus, during this study, it was determined whether it is possible to grow the above-mentioned grasses by fertilizing with sewage sludge compost in low-yielding soils of a light texture without harming nature (the soil) and the quality of the grasses’ biomass as solid biofuel. Also, the potential for using *B. inermis* and *Ph. arundinacea* to clear the soil of heavy metals was explored. There are no similar studies in Lithuania, and there are very few in Europe and the world.

## 2. Results and Discussion

### 2.1. Biomass Dry Matter (DM) Yield of Perennial Herbaceous Plants

As mentioned earlier, *Bromopsis inermis* Leyss. variety ‘*Galinda*’ and *Phalaroides arundinacea* L. variety ‘*Alaušas*’ are best adapted to the light soils and climatic conditions of southeastern Lithuania. The varieties are characterized by a high yield of green mass, dry matter, and seeds. According to the literature data, *B. inermis* variety ‘*Galinda*’ can produce up to 14.2 t/ha of biomass DM yield [25,41]. However, when the grass is cut only once per season, the dry matter yield of *B. inermis* biomass reaches 5–6 t/ha [42]. If the biomass of *Ph. arundinacea* variety ‘*Alaušas*’ is cut up to twice per season, a biomass yield of 5.0–15.9 t/ha DM can be obtained [24,42,43]. In the described experiment, grasses were cut once per season. Perhaps, because of this, lower yields of DM biomass from these plants were determined. (Table 1). Also, the low-yielding, light-textured soils of the experiment (sandy loam Haplic Luvisol (LVha)) negatively affected the yield. Although the plants studied are relatively tolerant to this soil, the biomass DM yield of *B. inermis* was on average 0.83–7.19 t/ha and of *Ph. arundinacea*—0.40–8.75 t/ha.

*Ph. arundinacea* and *B. inermis* did not produce a significant biomass yield in the first year of cultivation. Therefore, the first year’s biomass yield was not included in the biomass yield accounting. In the following years, the root system of the grasses developed even more, the plants grew stronger, and each year produced a larger biomass DM yield. Even in unfertilized fields, the biomass DM yield of *Ph. arundinacea* and *B. inermis* increased every year (Table 1).

The variance analysis of the 2015 biomass DM yield of the investigated perennial grasses revealed a significant impact of factor A (species of plant), factor B (fertilization), and the interaction of the A and B factors (Table 1). The variance analysis of the 2016 grasses biomass DM yield revealed a significant impact of only factor B (fertilization), and 2017—a significant impact of factor A (species of plant) and factor B (fertilization) (Table 1).

In 2015, *B. inermis* had a significantly higher biomass DM yield (1.29 t/ha on average) than *Ph. arundinacea* (on average 0.67 t/ha) (Table 1). When evaluating the interaction of the A (species of plant) and B (fertilization) factors, it was found that, in 2015, the biomass yield of DM of unfertilized and fertilized *B. inermis* was significantly higher than the biomass yield of DM of unfertilized and fertilized *Ph. arundinacea* (*p* < 0.05). *B. inermis* fertilized with mineral fertilizers had the highest biomass DM yield in 2015 (*p* < 0.01). In 2016, no significant difference was found between the yields of the investigated grasses. The biomass yield of DM was similar for both herbaceous plants. However, in 2017, *Ph. arundinacea* had a significantly higher DM biomass yield (average 3.67 t/ha) than *B. inermis* (average 2.91 t/ha). In other words, in the first harvest year (2015), *B. inermis* formed a higher biomass DM yield, but in the third harvest year, it was overtaken by *Ph. arundinacea*. The total biomass yield of DM in 2015, 2016, and 2017 for *Ph. arundinacea* and *B. inermis* was similar (on average 6.64 and 6.59 t/ha, respectively).

It was found that mineral fertilizers had a significant positive influence on the DM biomass yield of both studied perennial grass species in all harvest years (*p* < 0.05) (Table 1). However, the effect of sewage sludge compost was not so pronounced. Only the highest rate of SSC fertilization (125 t/ha) usually significantly increased the yield of grass biomass (*p* < 0.05). Analysis of the interaction between factors A (species of plant) and B (fertilization) showed that, in 2015 and 2017, fertilization with SSC did not significantly increase the biomass DM yield of either *B. inermis* or *Ph. arundinacea* (*p* > 0.05). In 2016, fertilization with 125 t/ha SSC significantly increased the biomass DM yield of both *B. inermis* and *Ph. arundinacea* (*p* < 0.05). When evaluating the interaction of the A and B factors, it was also found that fertilization with 125 t/ha SSC significantly increased the total biomass DM yield of *Ph. arundinacea* (*p* < 0.05) only (Table 1).

### 2.2. Plant Biomass Quality Parameters Which Determine Their Quality as a Solid Biofuel

The water content of energy plant biomass is a very important indicator that determines the quality of biomass as a solid biofuel. It, like other indicators of biomass quality, was evaluated in the fourth year of cultivation, at the end of the experiment in 2017. The lower the water content (%), the better the quality of the biomass, because less energy and costs are needed to dry such biofuel. In addition, the storage of moist fuel creates problems with its safety since moist biofuels can be spoiled by pathogens.

**Figure 1:**
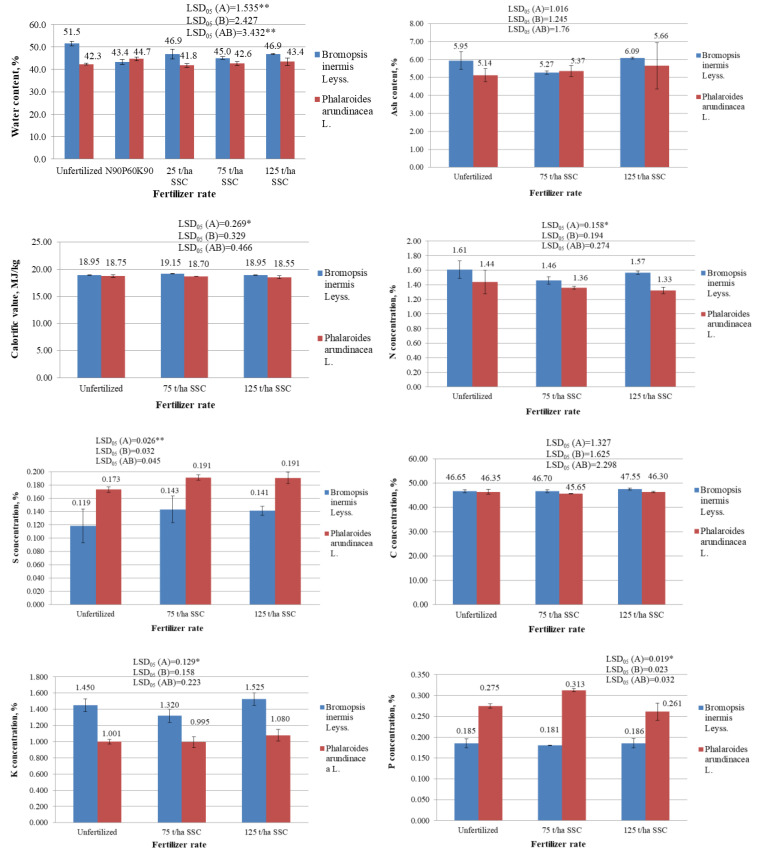
The biomass quality parameters of variously fertilized *Bromopsis inermis* Leyss. and *Phalaroides arundinacea* L., which determine its quality as a solid biofuel (2017 year). * and ** represent the statistically significant impact of the A factor, the B factor, or the interaction of the A and B factors at the 0.05 and 0.01 probability levels. LSD_05_—least statistically significant difference between variants of the experiment at the 0.05 probability level (*n* of water content = 3; *n* of other parameters = 2). Values are the mean ± SE (*n* of water content = 3; *n* of other parameters = 2).

During the research, the variance analysis of the water content of grass biomass revealed a significant impact of factor A (species of plant) and the interaction of the A and B (fertilization) factors. The water content in the biomass of *Ph. arundinacea* in 2017 was significantly lower (41.8–44.7%) than in the biomass of *B. inermis* (43.4–51.5%) (*p* < 0.05) (Figure 1). Factor B (fertilization) had no significant influence on the water content of the herbaceous plant biomass (*p* > 0.05). When evaluating the interaction of the A (species of plant) and B (fertilization) factors, it was found that the water content of the biomass of unfertilized *B. inermis* was significantly higher than that fertilized with mineral fertilizers, or 25, 75, and 125 t/ha SSC (*p* < 0.05). The use of fertilizers did not significantly affect the water content of the *Ph. arundinacea* biomass (*p* > 0.05).

The calorific value (MJ/kg) is the most important indicator describing the quality of energy plant biomass as a solid biofuel. The variance analysis of the calorific value of the plant biomass revealed a significant impact of factor A (species of plant); the calorific value of *B. inermis* biomass (19.02 MJ/kg) was significantly higher than that of *Ph. arundinacea* (18.67 MJ/kg) (*p* < 0.05). The calorific value of the investigated grasses was not significantly dependent on fertilization with SSC (Figure 1).

The amount of nitrogen (N) in the biomass of energy plants is an important indicator in terms of emissions [44]. Burning plants releases NO_x_ into the atmosphere. The amount of nitrogen emissions depends not only on the combustion temperature, the time spent in the high-temperature zone, and the oxygen concentration [45], but also on the N concentration in the fuel. The less nitrogen there is in the biomass of energy plants, the less the environment is polluted during combustion. The concentration of nitrogen in the biomass may depend on the variety or species of plants, as well as the type of fertilizer and the rate of application of fertilizers.

Potassium is one of the main components that determines the melting point of ash and the scale of surfaces. Potassium is also the main element forming volatile ash, which comes out with smoke in the form of aerosols during combustion. High concentrations of potassium in the biomass of energy plants promote the formation of fine ash, and the formation of KCl causes slagging problems. However, it is believed that K can be removed from biofuel by washing it with water or slightly acidic solutions [46].

The variance analysis of biomass nitrogen (N) concentrations and potassium (K) concentrations also revealed a significant impact of factor A (species of plant) only (Figure 1). The *B. inermis* biomass contained significantly more nitrogen (1.46–1.61%) than the *Ph. arundinacea* biomass (1.33–1.44%) (*p* < 0.05). (Figure 1). Also, the *B. inermis* biomass contained significantly more potassium (1.320–1.525%) than the *Ph. arundinacea* biomass (0.995–1.080%) (*p* < 0.05) (Figure 1). Such high concentrations of N and K in the biomass of the investigated grasses greatly reduce the quality of their biomass as a solid biofuel. The concentrations of nitrogen and potassium in the biomass of the studied grasses were not significantly dependent on fertilization with SSC (*p* > 0.05) (Figure 1). However, Antonkiewicz et al. [33] determined that the N and K concentrations in the biomass of the *Ph. arudinacea* ‘Bamse’ cultivar significantly increased after fertilizing this grass with municipal sewage sludge.

The sulfur concentration in plant biomass is a very important indicator of biofuel quality in terms of emissions. During biomass burning, SO_2_ is released [44,46], and the concentration of SO_2_ in combustion products depends on the sulfur content of the burning material [44,45]. In addition, during biomass burning, SO_3_ can be released. When SO_3_ combines with water (H_2_O), sulfuric acid (H_2_SO_4_) can be formed [44,45]. The lower the sulfur content in energy plants, the less sulfuric acid (H_2_SO_4_) can be produced. This means that corrosion will affect boilers less and they will last longer.

Phosphorus (P) is one of the main components that determines the melting point of energy plant ash and the scale of surfaces. The less it is present in the biomass, the better the quality of the biomass. Buinevicius [46] argued that phosphorus can be effectively removed by washing the fuel with water.

The variance analysis of sulfur (S) and phosphorus (P) concentrations in the biomass of herbaceous plants revealed a significant impact of only factor A (species of plant). The *Ph. arundinacea* biomass contained significantly more sulfur (0.173–0.191%) and phosphorus (0.261–0.313%) than the *B. inermis* biomass (0.119–0.143% and 0.181–0.186%, respectively) (*p* < 0.05) (Figure 1). The concentrations of these parameters in the biomass of the investigated grasses were not significantly dependent on fertilization with SSC (*p* > 0.05).

Ash content is another very important quality indicator of plant biomass as a solid biofuel. The less ash produced in the process of burning energy plants, the longer the boilers’ service life can be. Also, the emission of solid particles increases during the combustion process. Carbon, along with hydrogen and sulfur, are the combustible elements that make up solid biofuels [47].

The ash content of the investigated plant biomasses and carbon (C) concentrations in plant biomasses were not significantly dependent on either the plant species or fertilization with SSC. (*p* > 0.05) The average ash content of the *B. inermis* biomass was 5.27–6.09% (Figure 1) and the *Ph. arundinacea* biomass ash content was on average 5.14–5.66%. Such ash content is very high and not very suitable for solid biofuel. The carbon (C) concentration in the *B. inermis* biomass was on average 46.65–47.55% and in the *Ph. arundinacea* biomass—45.65–46.35% (Figure 1).

### 2.3. Concentration of Heavy Metals in Plant Biomass, mg/kg

#### 2.3.1. Zinc (Zn) Concentration in Plant Biomass, mg/kg

Studies of the heavy metal concentrations in the biomass of energy plants are very important in order to find out which energy plants absorb the most of them. Such energy plants could be used to clean the soil from heavy metals. This is especially relevant when fertilizing plants with sewage sludge compost (SSC).

Zinc (Zn) is one of the most easily absorbed heavy metals by plants. On average, the above-ground biomass of beans and cereals can contain 15–60 mg/kg of this element [48]. In the absence of zinc, the normal growth and development of plants is disrupted and the chlorosis of plants may occur [49]. Plants absorb zinc contained in the soil solution, exchange cations, and organic complexes [50]. However, excessive Zn concentrations in the soil can hinder plant development [51]. In addition, calcium, phosphates, and copper weaken the uptake of zinc into plants [52].

**Figure 2:**
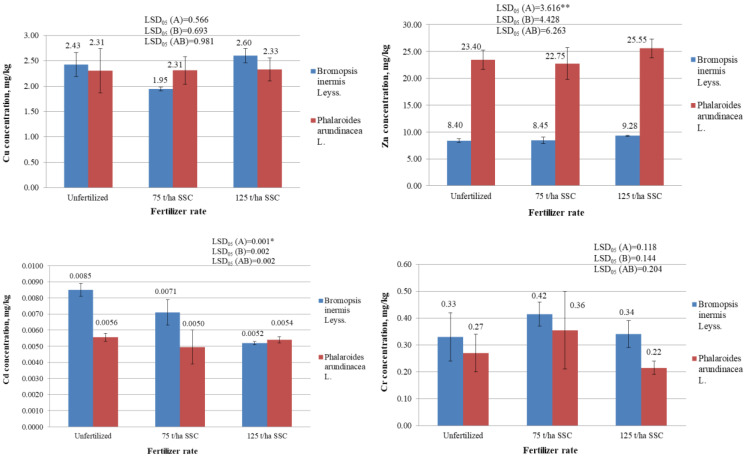

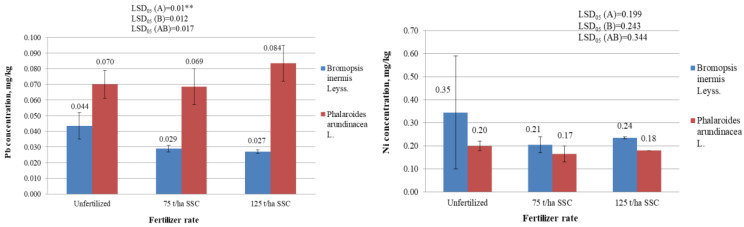
Concentrations of heavy metals (Cu, Zn, Cd, Cr, Pb, and Ni) in the biomass of variously fertilized *Bromopsis inermis* Leyss. and *Phalaroides arundinacea* L. (mg/kg) (2017 year). * and ** represent the statistically significant impact of the A factor, the B factor, or the interaction of the A and B factors at the 0.05 and 0.01 probability levels. LSD_05_—least statistically significant difference between variants of the experiment at the 0.05 probability level (*n* = 2). Values are the mean ± SE (*n* = 2).

The variance analysis of Zn concentration in the biomass of the investigated perennial grasses revealed a significant impact of factor A (species of plant) (Figure 2). The *Ph. arundinacea* biomass contained significantly more Zn (22.75–25.55 mg/kg) than the *B. inermis* biomass (8.40–9.28 mg/kg) (*p* < 0.05). Evaluating the interaction of the A (species of plant) and B (fertilization) factors, it was also found that the Zn concentration in the biomass of unfertilized and fertilized *Ph. arundinacea* was significantly higher than in the biomass of unfertilized and fertilized *B. inermis* (*p* < 0.05). This means that *Ph. arundinacea* is able to absorb Zn from the soil more than *B. inermis*. For this reason, *Ph. arundinacea* could potentially be used for Zn scavenging from soil. Rosikon et al. [53] also noted that *Ph. arundinacea* accumulates zinc in their biomass better than *Miscanthus giganteus* L.

The zinc concentration in the biomass of *B. inermis* and *Ph. arundinacea* did not change significantly depending on fertilization with SSC (*p* > 0.05).

#### 2.3.2. Cadmium (Cd) Concentration in Plant Biomass, mg/kg

The variance analysis of the cadmium (Cd) concentration in the biomass of the studied perennial grasses also revealed a significant impact of factor A (species of plant) (Figure 2). However, the Cd concentrations were significantly higher in the *B. inermis* biomass than in the *Ph. arundinacea* biomass (*p* < 0.05). An analysis of the interaction between the factors A (species of plants) and B (fertilization) also showed that the Cd concentration in the biomass of unfertilized and fertilized, with 75 t/ha SSC, *B. inermis* was significantly higher than in biomass of unfertilized and fertilized, with 75 t/ha SSC, *Ph. arundinacea* (*p* < 0.05). After fertilization with 125 t/ha SSC, the concentration of Cd in the *B. inermis* biomass decreased significantly (*p* < 0.05). This may have been because the sewage sludge compost increased the soil pH while reducing the cadmium mobility in soil and its availability to plants. However, the concentration of cadmium in the biomass of *Ph. arundinacea* did not change significantly after fertilization with sewage sludge compost. Rosikon et al. [53] also found that the fertilization of *Ph. arundinacea* with municipal sewage sludge did not cause a significant increase in the Cd concentrations in biomass in the first year of the experiment.

#### 2.3.3. Lead (Pb) Concentration in Plant Biomass, mg/kg

The study found that lead concentrations, as well as zinc concentrations, were significantly higher in the *Ph. arundinacea* biomass (0.069–0.084 mg/kg) than in the *B. inermis* biomass (0.027–0.044 mg/kg) (*p* < 0.05) (Figure 2). The variance analysis of the Pb concentration in the biomass of the studied perennial grasses revealed a significant impact of factor A (species of plant). This may mean that *Ph. arundinacea,* more than *B. inermis,* takes up Pb from the soil and is more suitable for use in soil lead removal. When evaluating the interaction of the A and B factors, it was found that the lead concentration in the biomass of unfertilized and fertilized *Ph. arundinacea* was significantly higher than in the biomass of unfertilized and fertilized *B. inermis* (*p* < 0.05). In addition, it was found that soil fertilization with 125 t/ha SSC significantly increased the lead concentration in the *Ph. arundinacea* biomass (*p* < 0.05). The concentration of lead in the *B. inermis* biomass did not change significantly after fertilization with SSC (*p* > 0.05).

#### 2.3.4. Copper (Cu), Chromium (Cr), and Nickel (Ni) Concentration in Plant Biomass, mg/kg

During the study, neither grass species nor fertilization with compost had a significant effect on the concentrations of Cu, Cr, and Ni in the grasses’ biomass (*p* > 0.05) (Figure 2). However, Rosikon et al. [53] found that fertilization of *Ph. arundinacea* with municipal sewage sludge increased Ni concentrations in biomass in the first year of the experiment.

The copper (Cu) concentration in the *B. inermis* biomass was on average 1.95–2.6 mg/kg, and in the *Ph. arundinacea* biomass—2.31–2.33 mg/kg. Copper (Cu) is an essential element in plant nutrition. It is included in the composition of many enzymes, increases their activity, and stimulates other physiological functions of plants. Copper from the soil, and accordingly from the sewage sludge compost, enters the plants gradually. There are studies that show that even if the concentration of copper in the soil is increased 12 times, it is increased only up to 2 times in the grains, tubers, straws, and leaves [49,51]. Perhaps, this is why there was no increase in the biomass copper concentration in the herbaceous energy plants due to SSC fertilization. In addition, it is likely that a similar situation could occur as with Cd; compost fertilization could have increased the pH, decreased the mobility of copper, and, therefore, reduced its uptake into the plants.

Higher concentrations of chromium (Cr) are harmful to plants, animals, and humans. Cr can cause chlorosis of young plant leaves and stop root growth [49]. Bergman W. [51] found that if up to 55 mg/kg of Cr accumulates in potato leaves (SM), the tips become dry. In this study, the Cr concentration in the biomass of *B. inermis* was on average 0.33–0.42 mg/kg, and in the biomass of *Ph. arundinacea*—0.22–0.36 mg/kg (Figure 2).

Nickel is needed in small amounts by plants and other organisms. However, higher concentrations of nickel have a negative impact on plants and animals. For example, when growing oats in sandy soil with a high nickel content, leaf necrosis occurs [49]. Bergman W. [51] found that when there is more than 100 mg/kg of nickel in the soil, the chlorosis of leaves of potatoes, beans, and cereals is observed. Plants of the *Poaceae* family are particularly sensitive to excess nickel [52]. During the described study, the nickel (Ni) concentration in the *B. inermis* biomass was on average 0.21–0.35 mg/kg (Figure 2). The concentration of nickel in the biomass of the *Ph. arundinacea* was 0.17–0.2 mg/kg.

### 2.4. Differences in Values of the Agrochemical Parameters of the Soil between the Years 2014 and 2017 

#### 2.4.1. Differences in Soil pH

Soil acidity (pH) determines the mobility and absorption of nutrients by plants, their physical properties, the biological activity of microorganisms, plant growth, and development [54]. Also, in the case of acidic soil, the mobility of heavy metals increases [55]; therefore, in Lithuania, it is prohibited to use all types and categories of sewage sludge in agriculture if the soil pH is <5.5 [56]. The soil pH of the described experiment was higher than 5.5 (pH 6.2–6.5), i.e., fertilization with SSC was possible.

**Figure 3:**
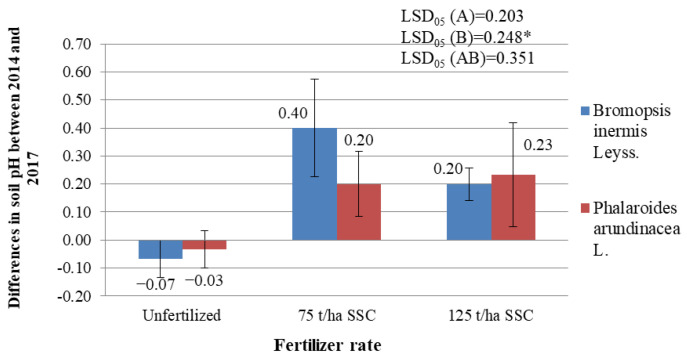
Differences in soil pH in the experiment of variously fertilized *Bromopsis inermis* Leyss. and *Phalaroides arundinacea* L. between 2014 and 2017. * represent the statistically significant impact of the A factor, the B factor, or the interaction of the A and B factors at the 0.05 probability level. LSD_05_—least statistically significant difference between variants of the experiment at the 0.05 probability level (*n* = 3). Values are the mean ± SE (*n* = 3).

The soil pH was analyzed prior to the installation of the herbaceous plant experiment in 2014 and after four years of plant growth at the end of the experiment in 2017. The soil pH change over the four years of study was analyzed; the results are presented in Table S1 and Figure 3. It was found that in unfertilized fields, the soil became more acidic (pH decreased by 0.03–0.07) (Figure 3), and in the fields that were fertilized with the highest investigated rates of SSC (75 t/ha and 125 t/ha), the soil pH increased (0.20–0.40) (Figure 3). SSC significantly increased the soil pH over the years studied. Although the variance analysis of the soil pH change revealed a significant impact of factor B (fertilization), SSC significantly increased the pH increase during the study years only in the soil where *B. inermis* (*p* < 0.05) grew. An analysis of the interaction between factors A (species of plant) and B also showed that the increase in soil pH where *Ph. arundinacea* was grown also increased, but not significantly (*p* > 0.05). Changes in the pH of the soil where the *B. inermis* grew over the years of research did not significantly differ from the changes in the pH of the soil where *Ph. arundinacea* grew.

#### 2.4.2. Differences in Mobile Phosphorus (P_2_O_5_) Concentrations in the Soil, mg/kg

**Figure 4:**
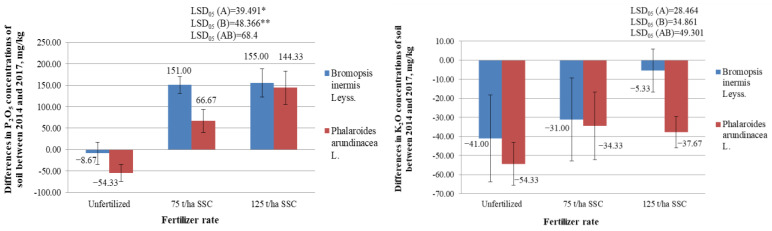
Differences in the mobile phosphorus (P_2_O_5_) and mobile potassium (K_2_O) concentrations in the experimental soil of variously fertilized *Bromopsis inermis* Leyss. and *Phalaroides arundinacea* L. between 2014 and 2017 (mg/kg). * and ** represent the statistically significant impact of the A factor, the B factor, or the interaction of the A and B factors at the 0.05 and 0.01 probability levels. LSD_05_—least statistically significant difference between variants of the experiment at the 0.05 probability level (*n* = 3). Values are the mean ± SE (*n* = 3).

During the research, the soil mobile phosphorus (P_2_O_5_) concentrations were determined before the installation of the experiment and at the end of the experiment. However, as in the case of pH, the change in the mobile phosphorus (P_2_O_5_) concentrations during the research years was analyzed because that was the most important thing. The variance analysis of the change in the concentration of mobile phosphorus revealed a significant impact of factor A (species of plant) and factor B (fertilization) (Table S2). It was found that after four years of the experiment, the concentration of P_2_O_5_ in the unfertilized soil decreased, while in the soil that was fertilized with sewage sludge compost, it significantly increased (Table S2). Fertilization with sewage sludge compost at rates of 75 t/ha and 125 t/ha significantly increased the increase in P_2_O_5_ concentrations in the soil (*p* < 0.05) (Figure 4). Analysis of the interaction between factors A and B also showed that fertilization with sewage sludge compost significantly increased the increase in P_2_O_5_ concentrations in the soil where both *B. inermis* and *Ph. arundinacea* grew (*p* < 0.05). It was also found that the concentration of mobile phosphorus increased less in the soil where *Ph. arundinacea* grew than in the soil where *B. inermis* grew (*p* < 0.05). This may mean that *Ph. arundinacea* can absorb more P_2_O_5_ from the soil and remains significantly less in the soil.

#### 2.4.3. Differences in Mobile Potassium (K_2_O) Concentrations in the Soil, mg/kg

It was found that the K_2_O concentrations in the soil decreased significantly after four years of research (Table S3 and Figure 4). In the soil where *B. inermis* grew, and which was fertilized with 125 t/ha SSC, K_2_O concentrations decreased significantly less (*p* < 0.05). That is, fertilization with 125 t/ha of sewage sludge compost significantly increased the K_2_O concentrations in the soil where the *B. inermis* grew. No significant difference was found between the concentrations of mobile potassium in the soil where *B. inermis* grew and the concentrations of mobile potassium in the soil where the *Ph. arundinacea* grew.

#### 2.4.4. Differences in Total Nitrogen (N_total_) (%) and in Organic Carbon (C_org._) (%) Concentrations in the Soil

**Figure 5:**
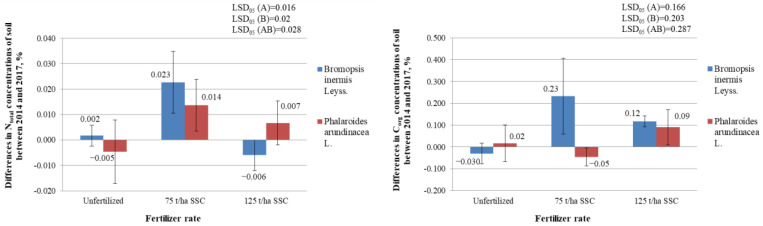
Differences in the total nitrogen (N_total_) (%) and in organic carbon (C_org._) (%) concentrations in the experimental soil of variously fertilized *Bromopsis inermis* Leyss. and *Phalaroides arundinacea* L. between 2014 and 2017. LSD_05_—least statistically significant difference between variants of the experiment at the 0.05 probability level (*n* = 3). Values are the mean ± SE (*n* = 3).

The study found that neither the sewage sludge compost fertilizer nor the plant species grown in this soil had a statistically significant effect on the concentration of total nitrogen (N_total_) and organic carbon (C_org._) in the soil (*p* > 0.05) (Tables S4 and S5 and Figure 5).

### 2.5. Differences in Concentrations of Heavy Metals (Cu, Zn, Cd, Cr, Pb, and Ni) in the Soil between the Years 2014 and 2017, mg/kg

#### 2.5.1. Differences in Concentrations of Copper (Cu) in the Soil, mg/kg

As already mentioned, the concentrations of heavy metals in the soil were determined before the installation of the experiment and after four years, at the end of the experiment. Changes in heavy metal concentrations in the soil that occurred during the four years of the experiment were analyzed. The main objective was to determine how fertilization with SSC affected the concentrations of heavy metals in the soil, and which plant (*B. inermis* or *Ph. arundinacea*) better cleans the soil of heavy metals.

**Figure 6:**
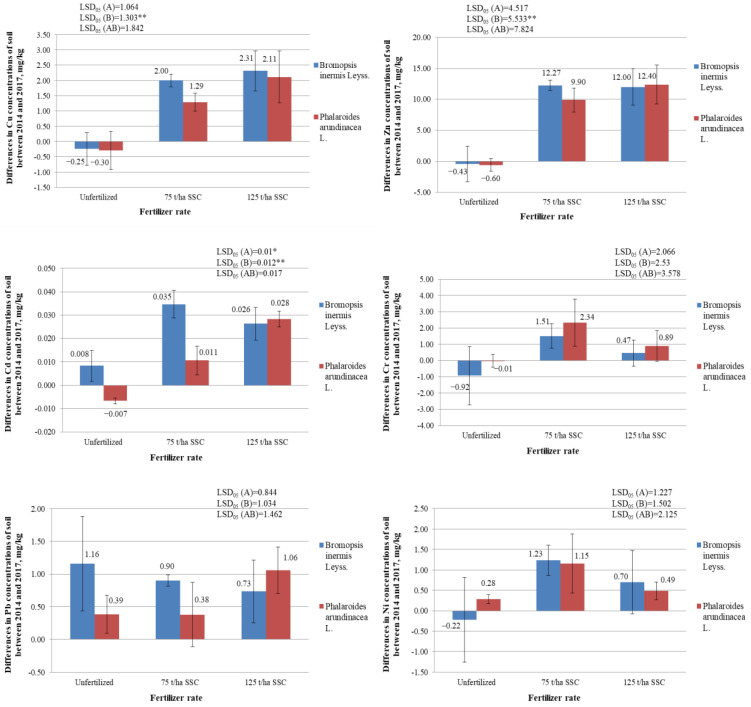
Differences in the concentrations of heavy metals (Cu, Zn, Cd, Cr, Pb, and Ni) in the soil in the experiment of variously fertilized *Bromopsis inermis* Leyss. and *Phalaroides arundinacea* L. between 2014 and 2017 (mg/kg). * and ** represent the statistically significant impact of the A factor, the B factor, or the interaction of the A and B factors at the 0.05 and 0.01 probability levels. LSD_05_—least statistically significant difference between variants of the experiment at the 0.05 probability level (*n* = 3). Values are the mean ± SE (*n* = 3).

It was found that the variance analysis of the change in copper (Cu) concentration revealed a significant impact of factor B (fertilization) (Figure 6). The 75 and 125 t/ha rates of SSC significantly increased the Cu concentrations in the soil where *B. inermis* and *Ph. arundinacea* grew (*p* < 0.05) (Figure 6; Table S6). An analysis of the interaction between factors A (species of plant) and B also confirmed this tendency. At the beginning of the experiment, there was an average of 6.00–6.47 mg/kg of Cu in the soil (Table S6). The maximum allowable concentration (MAC) of Cu in Lithuania for sewage sludge of the II category is 300–1000 mg/kg [56]. After fertilization with SSC containing 142 mg/kg of Cu, the Cu concentrations in the soil became 6.00–8.53 mg/kg. These concentrations did not exceed the maximum allowable concentration of Cu in soil in Lithuania—50 mg/kg.

Copper is one of the more mobile elements in nature, especially in an acidic medium. However, this element is immobile in an alkaline medium. At a soil pH of 5.4–6.1, Cu precipitates as hydroxides and phosphates [49]. The soil pH of the described experiment was 6.20–6.70. As a result, there should have been little mobile Cu in the soil. However, it may still have been mobile because, even though the Cu concentration in the soil increased after SSC was applied, the Cu concentration in the grass biomass did not change significantly. The changes in the Cu concentrations in the soil after four years of the study were not significantly dependent on the cultivated plant species (*p* > 0.05) (Figure 6; Table S6).

#### 2.5.2. Differences in Concentrations of Zinc (Zn) in the Soil, mg/kg

High concentrations of zinc (Zn) accumulated in the soil can inhibit plant growth. Bergman W. [51] found that when the total Zn in the soil is 1000 mg/kg, the yield of winter wheat is strongly reduced, and when Zn concentration is 1500 mg/kg, there is no yield. Dudka et al. [57] argued that Zn concentrations in the soil of up to 200–300 mg/kg are not harmful to plants. However, from an environmental and legal point of view, such high concentrations of Zn in the soil are unacceptable. The following soil Zn concentrations were determined at the beginning of the described experiment: 18.10–22.20 mg/kg (Table S7). The concentration of Zn in the SSC was 710 mg/kg, while the MAC in Lithuania for sewage sludge of the II category is 800–2500 mg/kg [56]. After fertilization with SSC, Zn concentrations in the soil (28.00–32.57 mg/kg) did not exceed the MAC 160 mg/kg. However, a variance analysis of the change in Zn concentration revealed the significant impact of factor B (fertilization) (Figure 6). The 75 and 125 t/ha rates of SSC significantly increased the Zn concentrations in the soil where *B. inermis* and *Ph. arundinacea* grew (*p* < 0.05). An analysis of the interaction between factors A (species of plant) and B also confirmed this tendency. Changes in the Zn concentrations in the soil were not significantly dependent on the species of grass grown in this soil (*p* > 0.05) (Figure 6).

#### 2.5.3. Differences in Concentrations of Cadmium (Cd) in the Soil, mg/kg

A variance analysis of the change in cadmium (Cd) concentration revealed a significant impact of factor A (species of plant) and factor B (fertilization) (Figure 6). SSC significantly increased the Cd concentrations in the soil (*p* < 0.05). When evaluating the interaction of the A and B factors, it was found that 75 t/ha and 125 t/ha rates of SSC significantly increased the Cd concentrations in the soil where both the *B. inermis* and *Ph. arundinacea* grew (*p* < 0.05). In the soil where *Ph. arundinacea* grew, Cd concentrations increased significantly less during the four years of the study than in the soil where *B. inermis* grew (*p* < 0.05).

Bergman W. [51] conducted studies and found that with a Cd concentration in the soil of 100–300 mg/kg, wheat yield decreases and sugar beets almost completely disappear. Potato leaves turn yellow if there is 100 mg/kg of cadmium in the soil. However, such high concentrations of Cd are rare in soil. At the beginning of the described study, the Cd concentrations were 0.047–0.059 mg/kg (Table S8). After fertilization with SSC, the Cd concentrations were 0.069–0.087 mg/kg and did not exceed the Lithuanian MAC—1.0 mg/kg [56]. The concentration of Cd in the SSC used in the experiment was 1.81 mg/kg, while the Lithuanian MAC for sewage sludge of the II category is 1.5–5 mg/kg.

#### 2.5.4. Differences in Concentrations of Chromium (Cr) in the Soil, mg/kg

It was found that fertilization with SSC increased the concentration of chromium (Cr) in the soil. However, the differences in Cr concentrations in unfertilized and fertilized soil were insignificant (*p* > 0.05) (Figure 6, Table S9). It was not found that one plant species under study removed chromium from the soil more than another plant species.

During the described experiment, the soil was fertilized with sewage sludge compost containing 40.5 mg/kg of Cr (the MAC of Cr in Lithuania for sewage sludge of the II category is 140–170 mg/kg) [56]. The initial soil Cr concentrations were 7.70–10.53 mg/kg (Table S9). After fertilization with SSC, the Cr concentrations were 9.60–10.53 mg/kg and did not exceed the MAC of Cr in Lithuania—50 mg/kg [56].

#### 2.5.5. Differences in Concentrations of Lead (Pb) in the Soil, mg/kg

In nature, lead (Pb) is found in compounds or soluble [49,58]. The most common lead compounds are lead sulfides (PbS) and sulfates (PbSO_4_) [59]. Lead begins to affect plants when it accumulates in the soil in large quantities—100–200 mg/kg [49]. It is especially harmful in acidic soils (pH < 5.0) [49]. As already mentioned, the soil pH ranged from 6.20 to 6.70 during the described study.

The initial concentrations of Pb in the soil of the experiment were 7.74–8.13 mg/kg (Table S10). After fertilization with SSC, the Pb concentrations were 8.30–9.19 mg/kg and did not exceed the MAC of 50 mg/kg in Lithuania [56]. The concentration of Pb in the SSC was 40.0 mg/kg, while the MAC in Lithuania for sewage sludge of the II category is 140–150 mg/kg. Changes in the Pb concentrations in the soil after four years of the study were not significantly dependent on the fertilization with SSC or on the cultivated plant species (*p* > 0.05) (Figure 6, Table S10).

#### 2.5.6. Differences in Concentrations of Nickel (Ni) in the Soil, mg/kg

Changes in the Ni concentrations in the soil after four years of the study were not significantly dependent on fertilization with sewage sludge compost or the plant species (*p* > 0.05) (Figure 6, Table S11). At the beginning of the experiment, the Ni concentrations in the soil were 5.90–7.29 mg/kg (Table S11). After fertilization with SSC, the Ni concentrations increased to 7.05–7.28 mg/kg but did not exceed the MAC of Ni in Lithuania (50 mg/kg) [56]. The concentration of Ni in the sewage sludge compost, which was used to fertilize the studied grasses, was 25.1 mg/kg. This did not exceed the MAC in Lithuania for sewage sludge of the II category (50–70 mg/kg).

## 3. Materials and Methods

### 3.1. Field Experiment

**Figure 7:**
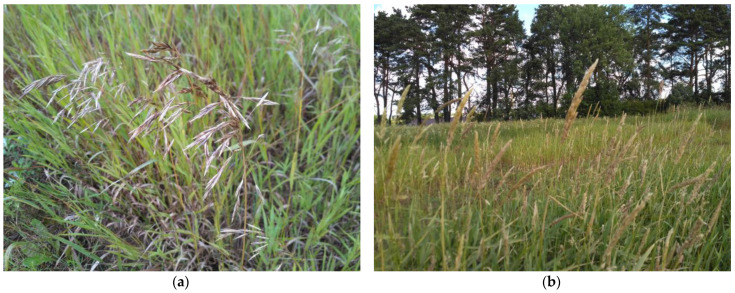
*Bromopsis inermis* Leyss. (variety ‘*Galinda*’) (**a**) and *Phalaroides arundinacea* L. (variety ‘*Alaušas*’) (**b**) at the experiment site at the Vokė Branch of the Institute of Agriculture of the Lithuanian Research Centre for Agriculture and Forestry (LAMMC).

The experiment was carried out at the Vokė Branch of the Institute of Agriculture of the Lithuanian Research Centre for Agriculture and Forestry (LAMMC) on a soil sandy loam Haplic Luvisol (LVha) [60] from 2014 to 2017 (Figure 7). The experimental design was a Split-Plot design (Table 2). The length of the field was 4 m. The width was 4 m. The area was 16 m^2^. Repetitions—4. Number of fields—40.

The seed rate of *Bromopsis inermis* Leyss. was 20 kg/ha. The width between the rows was 30 cm. The depth of seed insertion was 1.5–3 cm. After sowing, they were pressed. *Phalaroides arundinacea* L. was sown, like the *B. inermis*, in a pure crop. The seed rate was 10 kg/ha, the seed insertion depth—0.5–2 cm, and the width between the rows—30 cm.

Scheme of the field experiment:

Factor—A (species of plant)

1. *Bromopsis inermis* Leyss. (variety ‘*Galinda*’);
2. *Phalaroides arundinacea* L. (variety ‘*Alaušas*’).

Factor—B (fertilization)

1. Unfertilized (control);
2. N_90_P_60_K_90_ (90 kg/ha N, 60 kg/ha P_2_O_5_, 90 kg/ha K_2_O);
3. 25 t/ha SSC DM;
4. 75 t/ha SSC DM;
5. 125 t/ha SSC DM.

Mineral NPK fertilizers were added every year at the beginning of vegetation in spring. Sewage sludge compost was incorporated into the soil once in the spring before the experiment installation (2014). In 2014, all types of fertilizers were incorporated into the soil at a depth of 20 cm. Then, the mineral fertilizers were poured onto the soil surface every year (2015, 2016, and 2017).

### 3.2. Sewage Sludge Compost (SSC)

To fertilize the grasses, compost from the sewage sludge of the city of Vilnius was used. Sewage sludge compost was obtained from the company “Biastra Plius” in the spring of 2014. It was then analyzed before setting up the experiment. The following parameter values were determined in the SSC: N_total_ 1.34%, P_total_ 0.90%, K_total_ 0.37%, organic matter 29.3%, C_org._ 17.8%, and pH_KCl_ 7.1.

Also, the following heavy metals were determined in the SSC samples by the standardized method of atomic absorption spectrometry [61]: copper (Cu), zinc (Zn), cadmium (Cd), chromium (Cr), lead (Pb), and nickel (Ni). The heavy metal concentrations were as follows: lead (Pb) 40.0 mg/kg, chromium (Cr) 40.5 mg/kg, nickel (Ni) 25.1 mg/kg, copper (Cu) 142 mg/kg, zinc (Zn) 710 mg/kg, and cadmium (Cd) 1.81 mg/kg.

A mineral nitrogen fertilizer rate of 90 kg/ha was applied to the perennial grasses. The minimum sewage sludge compost rate used in the experiment was calculated according to the amount of nitrogen for four years. If the annual nitrogen of the mineral fertilizers is 90 kg/ha, then it is four times higher than the compost—360 kg/ha. It was about 25 t/ha of SSC DM. There is no legal act describing the requirements for fertilization with SSC in Lithuania; therefore, it was decided to follow the legal acts describing sewage sludge norms. At the time of the experiment, in accordance with LAND 20-2005 [56], the maximum allowable amount of sewage sludge spread on the fields was 33 t/ha of DM per year. Thus, 132 t/ha SSC DM was allowed to be incorporated into the soil for four years for fertilizing energy plants. For the described experiment, the maximum rate of application of fertilizers was chosen close to the maximum permissible rate—125 t/ha SSC DM. The fertilizer rate of 75 t/ha SSC DM was chosen as an intermediate between the minimum and maximum rates.

### 3.3. Plant Biomass Parameters

#### 3.3.1. Biomass Dry Matter (DM) Yield of Perennial Herbaceous Plants

*Ph. arundinacea* and *B. inermis* did not produce a significant biomass yield in the first year of cultivation. As a result, it was decided not to include the first year’s harvest in the biomass harvest accounting. After that, the effects of SSC and mineral fertilizers on biomass formation of bioenergy herbaceous plants were monitored annually. At the end of the growing season (end of September—mid-October) in 2015, 2016, and 2017, the green mass yield of grasses was harvested and weighed. The dry matter (DM) biomass yield was also subsequently determined.

#### 3.3.2. Plant Biomass Quality Parameters Which Determine Its Quality as a Solid Biofuel

Samples of plant biomass, to determine their appropriateness for solid biofuel, were taken in the fourth year of the study before biomass harvesting (at the end of September 2017). Biomass samples were tested for [62,63]: water content, % [64] (weight method); calorific value, MJ kg^–1^; ash content, %; nitrogen (N) concentration; sulfur (S) concentration; carbon (C) concentration; potassium (K) concentration, and phosphorus (P) concentration.

The calorific value, MJ/kg [65] (direct burning) was determined with a Calorimeter C 2000 (IKA, Staufen, Germany). The analysis was carried out using a special methodology [66,67].

The ash content and % were determined using the weight method (ash content of dry mass = ash weight/dry sample weight × 100) [66].

The nitrogen (N), sulfur (S), and carbon (C) concentrations were analyzed with the Dumas method using a fully automatic Vario EL III analyzer (Elementar, Langenselbold, Germany). Samples of biomass weights (0.5 mg) were weighed in tin foil and put in the autosampler. The biomass samples were heated in a high-temperature furnace and rapidly combusted at about 1000 °C. The remaining elements were measured in a thermal conductivity detector (TCD) without any reference gas flow. The data management (input/output) was performed via computer [67].

The potassium (K) concentration was determined by atomic absorption spectrometry method using an atomic absorption analyzer Aanalyst 200 (PerkinElmer, Waltham, MA, USA) [67].

The phosphorus (P) concentration was determined spectrophotometrically at a wavelength of 430 nm with an atomic emission spectrophotometer Perkin Elmer Optima 2100 [67].

#### 3.3.3. Heavy Metals in Plant Biomass

Samples of plant biomass for the analysis of heavy metals were taken during the fourth year of research in 2017 at the end of September. In them, using the standardized method of atomic absorption spectrometry [61], the following concentrations were determined: copper (Cu), zinc (Zn), cadmium (Cd), chromium (Cr), lead (Pb), and nickel (Ni).

Heavy metals, mg/kg, were measured by standardized atomic absorption spectrometry using an AA spectrometer with an air–acetylene flame Aanalyst 200 or a graphite furnace Aanalyst 600 (PerkinElmer, Waltham, MA, USA). The biomass samples were burned at 550 °C to ashes wherein the ash content was calculated. A total of 0.5 g of ash was then mineralized with 3 mL of 65% nitric acid (HNO_3_) (Honeywell, No. 30709) and 9 mL of 37% hydrochloric acid (HCl) (Honeywell, No. 30721) in a mineralisator Gerhardt Analytical System (type KT (-L) 20s, Konigswinter, Germany). The solution obtained after wet mineralization was filtered through a 0.8 μm filter, diluted to the mark in a 50 mL flask with deionized water (water deionization system Purite Analyst 320, Purite, Thame, UK), and shaken. If necessary, the solution was diluted. Subsequently, the total heavy metals concentrations (mg/L) were determined and converted to concentrations of the analyzed element (mg/kg) according to their proportions.

### 3.4. Soil Parameters

Soil samples were collected before the installation of the experiment (spring 2014) and at the end of the experiment (end of September 2017) at a depth of 0–20 cm. Later, in the laboratory, the agrochemical parameters were determined in these samples. All plant residues were carefully removed before grinding the soil samples. The soil was milled until the entire fraction passed through a sieve with a sufficiently fine mesh (1–2 mm).

The pH_KCl_ was determined by the potentiometric method (LST ISO 10390:2005) [68] using automatic pH meter (Labfit AS-3010D, Labfit Pty Ltd., Bayswater, WA, Australia). A pH analysis was performed using a 1:5 (vol/vol) soil suspension in 1 M KCl (Honeywell, No. 12636). The mixture was shaken for 60 min and left to sit for 1 h. The pH of the suspension was measured at 20 ± 2 °C with a pH meter and while stirring [68].

Mobile phosphorus (P_2_O_5_), mg/kg, and potassium (K_2_O), mg/kg, were extracted using a 1:20 (wt/vol) soil suspension of ammonium lactate-acetic acid extractant (pH 3.7). The suspension was shaken for 4 h. Mobile P_2_O_5_ was determined in the extract using ammonium molybdate (Honeywell, No. 09880) via the spectrometric method with a Shimadzu UV 1800 spectrophotometer (Shimadzu Corporation, Kyoto, Japan). Mobile K_2_O was determined using flame emission spectroscopy with a JENWAY PFP7 flame photometer (London, UK) [69].

The total nitrogen (N_total_), % was determined by Kjeldahl mineralization and a distillation system (UDK 139 Semi-Automatic Kjeldahl Distillation Unit, VELP Scientifica, Usmate Velate (MB), Italy) (LST ISO 11261:1995) [70]. The method consists of heating a sample to 360–410 °C with concentrated sulfuric acid (H_2_SO_4_) (Honeywell, No. 30743), which decomposes the organic sample by oxidation to liberate the reduced nitrogen as ammonium sulfate. Hot concentrated sulfuric acid oxidizes carbon and sulfur. Hg_2_SO_4_ catalysts (Penta No. 25740) are added to make the digestion go faster. Na_2_SO_4_ or K_2_SO_4_ (Chempur, No. 118078707) is also added to increase the boiling point of H_2_SO_4_. Digestion is complete when the liquor is clarified with the release of fumes. The end of the condenser is dipped into the volume of HCl acid (Honeywell, No. 30721) and the sample solution is then distilled with a small amount of sodium hydroxide (NaOH) (Chempur, No. 118078707) [70].

Organic carbon (C_org._), %, was determined by the Turin method according to ISO 10694:1995 [71] with dry combustion and a total carbon analyzer Liqui TOC II (Elementar Analysensysteme GmbH, Langenselbold, Germany). The carbon in the soil is oxidized to carbon dioxide (CO_2_) by heating the soil to at least 900 °C in a stream of carbon-free synthetic air. To determine the organic carbon, carbonates are first removed using a hydrochloric acid solution (HCl) (Honeywell, No. 30721), 4 M. The organic carbon concentration was calculated by subtracting carbonate carbon from the total carbon concentration [71].

Also, the following heavy metals were determined in soil samples by the standardized method of atomic absorption spectrometry [61]: copper (Cu), zinc (Zn), cadmium (Cd), chromium (Cr), lead (Pb), and nickel (Ni). Heavy metals, mg/kg, were measured by standardized atomic absorption spectrometry using an AA spectrometer with an air–acetylene flame Aanalyst 200 or a graphite furnace Aanalyst 600 (PerkinElmer, Waltham, MA, USA). The soil samples were burned at 550 °C to ashes; the ash content was then calculated. A total of 0.5 g of ash was then mineralized with 3 mL of 65% nitric acid (HNO_3_) (Honeywell, No. 30709) and 9 mL of 37% hydrochloric acid (HCl) (Honeywell, No. 30721) in a mineralisator Gerhardt Analytical System (type KT (-L) 20s, Konigswinter, Germany). The solution obtained after wet mineralization was filtered through a 0.8 μm filter, diluted to the mark in a 50 mL flask with deionized water (water deionization system Purite Analyst 320, Purite, Thame, UK), and shaken. If necessary, the solution was diluted further. Subsequently, the total heavy metals concentrations (mg/L) were determined and converted to concentrations of the analyzed element (mg/kg) according to their proportions.

### 3.5. Statistical Analysis

The significant differences between the values of parameters of various variants (A factor—species of plant—and B factor—fertilization rate) were determined using the two-way analysis of variance method [72]. The Fisher test was used for the comparison of data [73]. Comparisons were performed using *p* < 0.05 and *p* < 0.01 probability levels (using Excel-based ANOVA (Microsoft Office Excel 2010)). Also, the results were presented as the arithmetic mean ± standard error (SE).

## 4. Conclusions

*Bromopsis inermis* Leyss. variety ‘*Galinda*’ and *Phalaroides arundinacea* L. variety ‘*Alaušas*’ formed a small total biomass DM yield (6.6 t/ha within three years) in the early years of cultivation. Also, the biomasses of these grasses had a very high ash content and concentrations of nitrogen and potassium, which greatly reduces the quality of the biomasses of these grasses as a solid biofuel. Although, the biomass of *B.inermis* had a higher calorific value (19.0–19.2 MJ/kg) and lower concentrations of sulfur and phosphorus in the biomass than *Ph. arundinacea* (*p* < 0.05). However, it may be appropriate to grow these grasses to clean the soil of heavy metals. *Ph. arundinacea* could potentially be used to clean the soil from Pb and Zn, and *B. inermis* could be used to clean the soil from Cd. More detailed research is needed to know for sure. During the experiment, fertilization with SSC did not significantly affect the quality parameters of the grass biomasses, which determine its quality as a solid biofuel. However, the compost performed poorly as a fertilizer. Fertilization with 125 t/ha SSC significantly increased the total biomass DM yield of *Ph. arundinacea* (*p* < 0.05) only. However, the biomass yield was still significantly lower than when fertilizing grasses with mineral fertilizers (*p* < 0.05). At the same time, using SSC as a fertilizer increased the concentration of heavy metals in the soil. For these reasons, it is not recommended to fertilize *B. inermis* and *Ph. arundinacea* with sewage sludge compost. However, when using SSC as a fertilizer, it is recommended to analyze the heavy metal concentrations in the SSC and to monitor the heavy metal concentrations in the soil before and after fertilization.

## Figures and Tables

**Table 1:** Biomass dry matter (DM) yield of variously fertilized *Bromopsis inermis* Leyss. and *Phalaroides arundinacea* L. (t/ha) (2015–2017 m).

Fertilizer Rate	Biomass Yield (DM)			Total Biomass Yield (DM) of All Years of the Experiment
	2015	2016	2017	
*Bromopsis inermis* Leyss. variety ‘*Galinda*’				
Unfertilized	0.83	1.27	1.78	3.88
N_90_P_60_K_90_	2.55	5.15	7.19	14.89
25 t/ha SSC DM	0.95	1.41	1.47	3.83
75 t/ha SSC DM	0.98	1.88	2.19	5.05
125 t/ha SSC DM	1.13	2.22	1.93	5.27
Average	1.29	2.38	2.91	6.59
*Phalaroides arundinacea* L. variety ‘*Alaušas*’				
Unfertilized	0.40	1.32	2.11	3.83
N_90_P_60_K_90_	1.41	4.42	8.75	14.58
25 t/ha SSC DM	0.47	1.51	1.94	3.92
75 t/ha SSC DM	0.44	1.96	2.43	4.83
125 t/ha SSC DM	0.63	2.27	3.13	6.03
Average	0.67	2.30	3.67	6.64
F_fact._				
Factor A	67.2 **	NI	9.88 **	NI
Factor B	45.48 **	46.75 **	95.61 **	78.85 **
Interaction A × B	3.02 *	NI	NI	NI
LSD_05_				
Species of plants (A)	0.155	0.38	0.506	0.975
Fertilization (B)	0.244	0.601	0.8	1.542
Interaction (A × B)	0.346	0.85	1.132	2.181

* and ** represent the statistically significant impact of the A factor, the B factor, or the interaction of the A and B factors at the 0.01 probability level. NI—there is no statistically significant impact. LSD_05_—least statistically significant difference between variants of the experiment at the 0.05 probability level (*n* = 4).

**Table 2:** Scheme of the experiment of *Bromopsis inermis* Leyss. and *Phalaroides arundinacea* L. (2014–2017).

		*Bromopsis inermis*			*Bromopsis inermis*			*Phalaroides arundinacea*			*Phalaroides arundinacea*
4 m	II repetition	2		IV repetition	1		II repetition	5		IV repetition	3
4 m		4			4			3			1
4 m		3			3			1			2
4 m		1			2			4			5
4 m		5			5			2			4
4 m	I repetition	3		III repetition	4		I repetition	4		III repetition	3
4 m		1			2			3			5
4 m		2			5			1			1
4 m		5			3			2			2
4 m		4			1			5			4
		4 m	2 m		4 m	2 m		4 m	2 m		4 m

1—Unfertilized (control); 2—N_90_P_60_K_90_; 3—25 t/ha SSC DM; 4—75 t/ha SSC DM; and 5—125 t/ha SSC DM.

## Data Availability

Data are contained within the article and Supplementary Materials.

## Supplementary Materials

The following supporting information can be downloaded at: https://www.mdpi.com/article/10.3390/plants12233939/s1, Table S1. Soil pH in the experiment of variously fertilized *Bromopsis inermis Leyss*. and *Phalaroides arundinacea* L.; Table S2. Mobile phosphorus (P_2_O_5_) concentrations in the soil of experiment of variously fertilized *Bromopsis inermis* Leyss. and *Phalaroides arundinacea* L., mg/kg; Table S3. Mobile potassium (K_2_O) concentrations in the soil of experiment of variously fertilized *Bromopsis inermis* Leyss. and *Phalaroides arundinacea* L., mg/kg; Table S4. Total nitrogen (N_total_) concentrations in the soil of experiment of variously fertilized *Bromopsis inermis* Leyss. and *Phalaroides arundinacea* L., %; Table S5. Organic carbon (C_org._) concentrations in the soil of experiment of variously fertilized *Bromopsis inermis* Leyss. and *Phalaroides arundinacea* L., %; Table S6. Copper (Cu) concentrations in the soil of experiment of variously fertilized *Bromopsis inermis* Leyss. and *Phalaroides arundinacea* L., mg/kg; Table S7. Zinc (Zn) concentrations in the soil of experiment of variously fertilized *Bromopsis inermis* Leyss. and *Phalaroides arundinacea* L., mg/kg; Table S8. Cadmium (Cd) concentrations in the soil of experiment of variously fertilized *Bromopsis inermis* Leyss. and *Phalaroides arundinacea* L., mg/kg; Table S9. Chromium (Cr) concentrations in the soil of experiment of variously fertilized *Bromopsis inermis* Leyss. and *Phalaroides arundinacea* L., mg/kg; Table S10. Lead (Pb) concentrations in the soil of experiment of variously fertilized *Bromopsis inermis* Leyss. and *Phalaroides arundinacea* L., mg/kg; Table S11. Nickel (Ni) concentrations in the soil of experiment of variously fertilized *Bromopsis inermis* Leyss. and *Phalaroides arundinacea* L., mg/kg.
