# Supplementary material for: Growing of Phalaroides arundinacea L. and Bromopsis inermis Leyss for Biofuel Using Sewage Sludge Compost as a Fertilizer
Source: Plants (Basel). 2023 Nov 22;12(23):3939. doi: 10.3390/plants12233939 (PMC10708169; doi:10.3390/plants12233939)
Supplement: Supplementary file 1 [file plants-12-03939-s001.zip › plants-2645767-supplementary.pdf]

plants

MDPI

Supplementary Materials

Table S1. Soil pH in the experiment of variously fertilized *Bromopsis inermis* Leyss. and *Phalaroides arundinacea* L.

**Fertilizer Rate**	**Soil pH**		**Difference in pH between 2014 and 2017**
	**2014**	**2017**	
Bromopsis inermis Leyss. variety '*Galinda*'			
Unfertilized	6.33	6.27	−0.07
75 t/ha SSC DM	6.20	6.60	0.40
125 t/ha SSC DM	6.50	6.70	0.20
Phalaroides arundinacea L. variety '*Alaušas*'			
Unfertilized	6.30	6.27	−0.03
75 t/ha SSC DM	6.47	6.67	0.20
125 t/ha SSC DM	6.37	6.60	0.23
$F_{fact.}$			
Factor A	-	-	NI
Factor B	-	-	5.39 *
Interaction A × B	-	-	NI
LSD_{05}			
Species of plants (A)	-	-	0.203
Fertilization (B)	-	-	0.248
Interaction (A × B)	-	-	0.351

* represent statistically significant impact of A factor; of B factor or of interaction of A and B factors at the 0.05 probability level. NI – there is no statistically significant impact. LSD_{05} – least statistically significant difference between variants of the experiment at the 0.05 probability level (n = 3).

Table S2. Mobile phosphorus (P_2O_5) concentrations in the soil of experiment of variously fertilized *Bromopsis inermis* Leyss. and *Phalaroides arundinacea* L., mg/kg.

Fertilizer Rate	P_2O_5 Concentrations of Soil		Difference in P_2O_5 Concentrations between 2014 and 2017
	2014	2017	
Bromopsis inermis Leyss. variety '*Galinda*'			
Unfertilized	225	217	−8.67
75 t/ha SSC DM	231	382	151.00
125 t/ha SSC DM	247	402	155.00
Phalaroides arundinacea L. variety '*Alaušas*'			
Unfertilized	266	212	−54.33
75 t/ha SSC DM	300	366	66.67
125 t/ha SSC DM	260	404	144.33
$F_{fact.}$			
Factor A	-	-	7 *
Factor B	-	-	38.33 **
Interaction A × B	-	-	NI
LSD_{05}			
Species of plants (A)	-	-	39.491
Fertilization (B)	-	-	48.366
Interaction (A × B)	-	-	68.4

* and ** represent statistically significant impact of A factor; of B factor or of interaction of A and B factors at the 0.05 and 0.01 probability levels. NI – there is no statistically significant impact. LSD_{05} – least statistically significant difference between variants of the experiment at the 0.05 probability level (n = 3).

Table S3. Mobile potassium (K_2O) concentrations in the soil of experiment of variously fertilized *Bromopsis inermis* Leyss. and *Phalaroides arundinacea* L., mg/kg.

**Fertilizer Rate**	**K_2O Concentrations of Soil**		**Difference in K_2O Concentrations between 2014 and 2017**
	**2014**	**2017**	
Bromopsis inermis Leyss. variety '*Galinda*'			
Unfertilized	138.00	97.00	−41.00
75 t/ha SSC DM	152.67	121.67	−31.00
125 t/ha SSC DM	131.33	126.00	−5.33
Phalaroides arundinacea L. variety '*Alaušas*'			
Unfertilized	149.33	95.00	−54.33
75 t/ha SSC DM	145.33	111.00	−34.33
125 t/ha SSC DM	145.33	107.67	−37.67
$F_{fact.}$			
Factor A	-	-	NI
Factor B	-	-	NI
Interaction A × B	-	-	NI
LSD_{05}			
Species of plants (A)	-	-	28.464
Fertilization (B)	-	-	34.861
Interaction (A × B)	-	-	49.301

NI – there is no statistically significant impact. LSD_{05} – least statistically significant difference between variants of the experiment at the 0.05 probability level (n = 3).

Table S4. Total nitrogen (N_{total}) concentrations in the soil of experiment of variously fertilized *Bromopsis inermis* Leyss. and *Phalaroides arundinacea* L., %.

Fertilizer Rate	N_{total} Concentrations of Soil		Difference in N_{total} Concentrations between 2014 and 2017
	2014	2017	
Bromopsis inermis Leyss. variety '*Galinda*'			
Unfertilized	0.093	0.095	0.002
75 t/ha SSC DM	0.092	0.115	0.023
125 t/ha SSC DM	0.091	0.085	−0.006
Phalaroides arundinacea L. variety '*Alaušas*'			
Unfertilized	0.089	0.084	−0.005
75 t/ha SSC DM	0.088	0.102	0.014
125 t/ha SSC DM	0.094	0.101	0.007
$F_{fact.}$			
Factor A	-	-	NI
Factor B	-	-	NI
Interaction A × B	-	-	NI
LSD_{05}			
Species of plants (A)	-	-	0.016
Fertilization (B)	-	-	0.02
Interaction (A × B)	-	-	0.028

NI – there is no statistically significant impact. LSD_{05} – least statistically significant difference between variants of the experiment at the 0.05 probability level (*n* = 3).

Table S5. Organic carbon ($C_{org.}$) concentrations in the soil of experiment of variously fertilized *Bromopsis inermis* Leyss. and *Phalaroides arundinacea* L., %.

**Fertilizer Rate**	**$C_{org.}$ Concentrations of Soil**		**Difference in $C_{org.}$ Concentrations between 2014 and 2017**
	**2014**	**2017**	
Bromopsis inermis Leyss. variety '*Galinda*'			
Unfertilized	0.91	0.88	−0.03
75 t/ha SSC DM	0.87	1.10	0.23
125 t/ha SSC DM	0.82	0.94	0.12
Phalaroides arundinacea L. variety '*Alaušas*'			
Unfertilized	0.84	0.86	0.02
75 t/ha SSC DM	0.91	0.86	−0.05
125 t/ha SSC DM	0.93	1.02	0.09
$F_{fact.}$			
Factor A	-	-	NI
Factor B	-	-	NI
Interaction A × B	-	-	NI
LSD_{05}			
Species of plants (A)	-	-	0.166
Fertilization (B)	-	-	0.203
Interaction (A × B)	-	-	0.287

NI – there is no statistically significant impact. LSD_{05} – least statistically significant difference between variants of the experiment at the 0.05 probability level (n = 3).

Table S6. Copper (Cu) concentrations in the soil of experiment of variously fertilized *Bromopsis inermis* Leyss. and *Phalaroides arundinacea* L., mg/kg.

Fertilizer Rate	Cu Concentrations of Soil		Difference in Cu Concentrations between 2014 and 2017
	2014	2017	
Bromopsis inermis Leyss. variety '*Galinda*'			
Unfertilized	6.47	6.22	−0.25
75 t/ha SSC DM	6.12	8.12	2.00
125 t/ha SSC DM	6.22	8.53	2.31
Phalaroides arundinacea L. variety '*Alaušas*'			
Unfertilized	6.31	6.01	−0.30
75 t/ha SSC DM	6.00	7.29	1.29
125 t/ha SSC DM	6.34	8.46	2.11
$F_{fact.}$			
Factor A	-	-	NI
Factor B	-	-	9.91 **
Interaction A × B	-	-	NI
LSD_{05}			
Species of plants (A)	-	-	1.064
Fertilization (B)	-	-	1.303
Interaction (A × B)	-	-	1.842

** represent statistically significant impact of A factor; of B factor or of interaction of A and B factors at the 0.01 probability level. NI – there is no statistically significant impact. LSD_{05} – least statistically significant difference between variants of the experiment at the 0.05 probability level (*n* = 3).

Table S7. Zinc (Zn) concentrations in the soil of experiment of variously fertilized *Bromopsis inermis* Leyss. and *Phalaroides arundinacea* L., mg/kg.

Fertilizer Rate	Zn Concentrations of Soil		Difference in Zn Concentrations between 2014 and 2017
	2014	2017	
Bromopsis inermis Leyss. variety '*Galinda*'			
Unfertilized	21.67	21.23	−0.43
75 t/ha SSC DM	18.40	30.67	12.27
125 t/ha SSC DM	18.87	30.87	12.00
Phalaroides arundinacea L. variety '*Alaušas*'			
Unfertilized	22.20	21.60	−0.60
75 t/ha SSC DM	18.10	28.00	9.90
125 t/ha SSC DM	20.17	32.57	12.40
$F_{fact.}$			
Factor A	-	-	NI
Factor B	-	-	16.08 **
Interaction A × B	-	-	NI
LSD_{05}			
Species of plants (A)	-	-	4.517
Fertilization (B)	-	-	5.533
Interaction (A × B)	-	-	7.824

** represent statistically significant impact of A factor; of B factor or of interaction of A and B factors at the 0.01 probability level. NI – there is no statistically significant impact. LSD_{05} – least statistically significant difference between variants of the experiment at the 0.05 probability level (n = 3).

Table S8. Cadmium (Cd) concentrations in the soil of experiment of variously fertilized *Bromopsis inermis* Leyss. and *Phalaroides arundinacea* L., mg/kg.

**Fertilizer Rate**	**Cd Concentrations of Soil**		**Difference in Cd Concentrations between 2014 and 2017**
	**2014**	**2017**	
Bromopsis inermis Leyss. variety '*Galinda*'			
Unfertilized	0.047	0.055	0.008
75 t/ha SSC DM	0.051	0.086	0.035
125 t/ha SSC DM	0.052	0.078	0.026
Phalaroides arundinacea L. variety '*Alaušas*'			
Unfertilized	0.057	0.051	−0.007
75 t/ha SSC DM	0.059	0.069	0.011
125 t/ha SSC DM	0.059	0.087	0.028
$F_{fact.}$			
Factor A	-	-	7.46 *
Factor B	-	-	13.08 **
Interaction A × B	-	-	NI
LSD_{05}			
Species of plants (A)	-	-	0.01
Fertilization (B)	-	-	0.012
Interaction (A × B)	-	-	0.017

* and ** represent statistically significant impact of A factor; of B factor or of interaction of A and B factors at the 0.05 and 0.01 probability levels. NI – there is no statistically significant impact. LSD_{05} – least statistically significant difference between variants of the experiment at the 0.05 probability level (n = 3).

Table S9. Chromium (Cr) concentrations in the soil of experiment of variously fertilized *Bromopsis inermis* Leyss. and *Phalaroides arundinacea* L., mg/kg.

Fertilizer Rate	Cr Concentrations of Soil		Difference in Cr Concentrations between 2014 and 2017
	2014	2017	
Bromopsis inermis Leyss. variety '*Galinda*'			
Unfertilized	10.53	9.61	−0.92
75 t/ha SSC DM	8.48	9.99	1.51
125 t/ha SSC DM	9.13	9.60	0.47
Phalaroides arundinacea L. variety '*Alaušas*'			
Unfertilized	9.56	9.54	−0.01
75 t/ha SSC DM	7.70	10.03	2.34
125 t/ha SSC DM	9.64	10.53	0.89
$F_{fact.}$			
Factor A	-	-	NI
Factor B	-	-	NI
Interaction A × B	-	-	NI
LSD_{05}			
Species of plants (A)	-	-	2.066
Fertilization (B)	-	-	2.53
Interaction (A × B)	-	-	3.578

NI – there is no statistically significant impact. LSD_{05} – least statistically significant difference between variants of the experiment at the 0.05 probability level (*n* = 3).

Table S10. Lead (Pb) concentrations in the soil of experiment of variously fertilized *Bromopsis inermis* Leyss. and *Phalaroides arundinacea* L., mg/kg.

**Fertilizer Rate**	**Pb Concentrations of Soil**		**Difference in Pb Concentrations between 2014 and 2017**
	**2014**	**2017**	
Bromopsis inermis Leyss. variety '*Galinda*'			
Unfertilized	7.74	8.90	1.16
75 t/ha SSC DM	7.79	8.69	0.90
125 t/ha SSC DM	7.96	8.70	0.73
Phalaroides arundinacea L. variety '*Alaušas*'			
Unfertilized	7.97	8.35	0.39
75 t/ha SSC DM	7.92	8.30	0.38
125 t/ha SSC DM	8.13	9.19	1.06
$F_{fact.}$			
Factor A	-	-	NI
Factor B	-	-	NI
Interaction A × B	-	-	NI
LSD_{05}			
Species of plants (A)	-	-	0.844
Fertilization (B)	-	-	1.034
Interaction (A × B)	-	-	1.462

NI – there is no statistically significant impact. LSD_{05} – least statistically significant difference between variants of the experiment at the 0.05 probability level (*n* = 3).

Table S11. Nickel (Ni) concentrations in the soil of experiment of variously fertilized *Bromopsis inermis* Leyss. and *Phalaroides arundinacea* L., mg/kg.

**Fertilizer Rate**	**Ni Concentrations of Soil**		**Difference in Ni Concentrations between 2014 and 2017**
	**2014**	**2017**	
Bromopsis inermis Leyss. variety '*Galinda*'			
Unfertilized	7.29	7.07	−0.22
75 t/ha SSC DM	5.90	7.13	1.23
125 t/ha SSC DM	6.58	7.28	0.70
Phalaroides arundinacea L. variety '*Alaušas*'			
Unfertilized	6.64	6.92	0.28
75 t/ha SSC DM	5.90	7.05	1.15
125 t/ha SSC DM	6.75	7.24	0.49
$F_{fact.}$			
Factor A	-	-	NI
Factor B	-	-	NI
Interaction A × B	-	-	NI
LSD_{05}			
Species of plants (A)	-	-	1.227
Fertilization (B)	-	-	1.502
Interaction (A × B)	-	-	2.125

NI – there is no statistically significant impact. LSD_{05} – least statistically significant difference between variants of the experiment at the 0.05 probability level (n = 3).
